# Removal of the Dental Pulp

**Published:** 1898-11

**Authors:** B. Holly Smith


### REMOVAL OF THE DENTAL PULP.[1]

BY DR. B. HOLLY SMITH.

"In proportion as medicine has grown scientific and skilful less dependence is placed on the therapeutic effects of drugs for the alleviation of human ills. Secondary effect has assumed greater importance, and possible evils attending administrations have outweighed the advantage of immediate relief.

"For more than half a century practitioners of dentistry have relied upon the application of arsenous acid to the dental pulp with a view to its destruction. Has any one found the exact limits of the destructive power of that agent? Has the use of this material been attended with no secondary evils? That pericemental disturbances do frequently occur can be stated without fear of dispute; that arsenic may be the cause has long been the thought of the writer, an opinion formed through observation of the contrast between teeth made pulpless through surgical procedure pure and simple and those from which the pulps have been

---

[1] Abstract of paper read before the National Dental Association, Omaha, 1898.

removed after the application of arsenical preparations. The well-known germicidal qualities of arsenic would indicate that no trouble should be expected from the contents of the tubuli which have been subjected to arsenical application and sealed from the chance of subsequent infection; yet trouble not infrequently does arise in after years, even where there is reasonable certainty that the apex was entirely closed.

"Why? Is it possible that a sleeping volcano has been located in the apical space by the application of arsenic? that the effect of the agent has not been limited to the confines of the pulp-canal and dentinal tubuli? that the area of tissue affected breaks down in seasons of depressed vitality, or becomes infected through the medium of the circulation? The apical opening may be infinitesimal, but it must be large in comparison with the tubuli in the dentine. Why may not the nerves and vessels in the apical space and a considerable area of the pericemental membrane have a share in the action of the arsenic? There is no positive evidence of a well-defined line of demarcation in the effect. Even after the agent passes the point where it has power to destroy, may it not deplete and incapacitate the tissues until danger is invited and may subsequently follow?"

Dr. Smith then quoted from Dr. J. Foster Flagg (1877), on the effects of arsenic taken into the circulation; from Arkövy, on the inflammation concomitant with the dying of the pulp; and from Dr. Burchard's recently published work, "Dental Pathology and Therapeutics," on the specific action of arsenic; from Dr. I. P. Wilson (1888), on its effects upon the cementum; and from Dr. Kirk (1898), on inflammatory action and bacterial invasion; thence deducing an explanation of the pathological expressions occurring in the apical space of teeth which have been treated with arsenic, drawing the conclusion that arsenic should no longer enjoy its universal popularity, but should give place to surgical procedure.

A change in this respect involves not so much the absence of other means as an aversion to change of practice, and the well-fixed habits of treating teeth without charge, giving two hours to filling a cavity with gold and five minutes to the destruction of a pulp. If, as we claim, we are dental surgeons,—not mere cavity-stoppers,—this is not right, and must be changed.

The conditions essential for pulp-removal by operative procedure were then considered, and the method of treatment outlined as follows:

"To the dental surgeon, as to the general surgeon, the necessity for operative procedure must be evident, and the field of operation be in as favorable condition as can be obtained. No effort should be made to remove a pulp in a high state of inflammation; employ antiphlogistic agents until irritation has subsided.

"Flood the cavity with a tepid solution of bicarbonate of soda. Then close the cavity with a pledget of cotton saturated in cocaine hydrochlorate for a few moments, avoiding pressure, and taking great care not to cause pain. Adjust a concave disk of vacuum-cavity material so that its edges will impinge upon the walls of the cavity and prevent pressure. Under this disk place a pledget of cotton saturated with oil of cloves. Cover with temporary stopping, paint the gum with aconite and iodine, equal parts, and dismiss patient until one or, preferably, two hours can be given to the operation of removal, when, if conditions are favorable, the pulp may be cocainized with the electric current and removed, using a minimum amount of current and a saturated solution of cocaine.

"If cataphoresis is not successful in the highest sense, supplant by a general anæsthetic, preferably nitrous oxide.

"When teeth containing living pulps are to be excised or ground down, advantage can be taken of the opportunity to remove the pulp under the influence of the shock of excision, which can be done with no pain. Cut the enamel with the disk or bur, leaving for the forceps only so much as will not require stress or violence. Having ready in the engine a cone-shaped bur, clean and sharp broach, etc., remove the pulp immediately by passing the broach quickly to the apex, twisting the pulp out in toto.

"The most inviting field for the surgical operation is found in single-root teeth, but it can be employed in the molars if the effort is painstaking. Surgery is not only more scientific, but is also proved to be more successful. A plea for this change in method should therefore not pass unheeded."

